# Characterization of individuals in home-based intensive care in Germany: data from a cross-sectional study

**DOI:** 10.3389/fneur.2026.1784277

**Published:** 2026-06-03

**Authors:** Patrick Hassl, Guido Faßbender, Martin Groß

**Affiliations:** 1 Linimed Gruppe, Jena, Germany; 2 MEDIAN Klinik Bad Tennstedt, Bad Tennstedt, Germany; 3 German Society for Out-of-Hospital Ventilation and Intensive Care (DIGAB) e.V., Göttingen, Germany; 4 Faculty of Medicine and Health Sciences, University of Oldenburg, Oldenburg, Germany

**Keywords:** home-based intensive care, home mechanical ventilation, out-of-hospital intensive care, tracheal tubes, tracheostomy, non-invasive ventilation

## Abstract

**Introduction:**

In Germany, approximately 23.000 individuals require home-based intensive care (HIC) provided by specialized nurses due to life-threatening conditions such as invasive ventilation, life-supporting noninvasive ventilation, or the presence of tracheal tubes requiring tracheobronchial suctioning. The current study’s objective was to characterize individuals receiving home-based intensive care from a private German provider to inform healthcare structures and clinical practice guidelines.

**Methods:**

On June 12, 2024, the quality management department of a private German home-based intensive care service collected cross-sectional data on all individuals receiving home-based intensive care.

**Results:**

Data from 851 individuals were collected, of which 511 (60%) were sixty years or older. Three hundred and thirty two individuals (39%) were female and 519 (61%) were male. Seven hundred and thirty four individuals (86%) were living in shared apartments and 117 (14%) lived at home with 24 h individual nursing care. Six hundred and eighty two individuals (80%) suffered from various neurological diseases, 116 (14%) from COPD, and 30 (4%) from oral, pharyngeal or laryngeal cancer. Twenty seven individuals (3%) had been admitted to HIC after a COVID-19 infection. Seven hundred and forty one individuals (89%) were tracheostomized, 220 (26%) needed invasive ventilation, 44 (5%) noninvasive ventilation and 14 (2%) hemodialysis.

**Conclusion:**

In Germany, neurological diagnoses and COPD are predominant in HIC. Most individuals are tracheostomized, and one in four requires invasive ventilation via tracheostomy. They also require multidisciplinary care by neurologists, pulmonologists, specialist nurses, respiratory therapists, speech-and-language therapists, physiotherapists, occupational therapists and other professionals. Neurological rehabilitation of individuals needing HIC should be established.

## Introduction

Home-based intensive care (HIC) (or: out-of-hospital intensive care) is provided to individuals who require the permanent presence of trained nurses. These individuals may have tracheal tubes, may require invasive ventilation via tracheostomy, or may depend on non-invasive ventilation that they are unable to manage independently. HIC can take place at the home of the individual as one-to-one nursing. It also can be provided in specialized shared appartements or nursing homes where nurses typically care for multiple individuals. The significance of sufficient staffing for patient safety is well established in the clinical setting, but corresponding data from the home-based intensive care setting are lacking (1).

In Germany, all nurses working in HIC must complete a curriculum consisting of 80 h of theoretical and 40 h of practical training. Additionally, nursing services are increasingly establishing professional supervision by specialist nurses or respiratory therapists. However, further training to become a respiratory therapist in Germany is not yet regulated by law.

The legal regulation of HIC is set out in Social Code Book V, paragraph 37c, and the subordinate regulation can be found in the HIC guideline of the Joint Federal Committee (Gemeinsamer Bundeausschuss, G-BA) (2). These regulations provide a compulsory framework comprising eligibility requirements, goals of care, specifications of healthcare provision, and quality criteria. The fact that individuals with tracheostomy, invasive ventilation or non-invasive ventilation may have the potential to be weaned from mechanical ventilation or to be decannulated is addressed through regular controls by qualified doctors. While pulmonologists may perform those controls medical specialists of other disciplines have to meet additional requirements regarding experience or qualification. Available evidence suggests that only a small proportion of individuals in HIC may have a potential for weaning from mechanical ventilation or decannulation. First estimates indicate that around 5% of individuals may show a short-term potential, while a further 19% could develop such a potential over time (3). The guideline further describes that healthcare professionals involved in HIC should work together as a network, hold team conferences, document treatment goals, integrate individuals and family caregivers and cooperate with institutions specialized in the primary diseases (2). Despite these regulations, the exact number of individuals requiring HIC is unknown, and their characteristics are poorly understood. However, the number of individuals in HIC in Germany is estimated to be about 23.000 (4). Individuals requiring HIC face substantial barriers to participation, but still may have a good or excellent quality of life, and some are even able to work in the primary labour market (5). However, there is no comprehensive structure for the HIC patients´ journey through multidisciplinary outpatient and inpatient health services to guarantee adequate rehabilitation.

A recently published analysis included routine data of a large health insurance more than 18.000 individuals receiving HIC from 2018 to 2022. However, data on diagnoses were not utilizable for health services planning or clinical practice. Additionally, the proportion of individuals with non-invasive ventilation was not provided. However, it was shown that 21% of all individuals needed tracheostomy invasive ventilation and that 62% had a tracheal tube, but did not need mechanical ventilation. The number of individuals receiving HIC at home declined over the years to 71% in the year 2022 versus 27% of the individuals being institutionalized—in 2% information was not provided (6). Half of the individuals requiring HIC died within 2 years but 30% survived more than 5 years (6). An analysis from the federal state of Hesse included more than 1,000 individuals, but did not report on diagnoses. Twenty nine percent received tracheostomy invasive ventilation, 10% needed noninvasive ventilation and 51% were tracheostomized without the need for mechanical ventilation (7). Fifty seven percent received HIC at home, 24% in nursing homes, 14% in shared appartements and 5% in other places (7). Another sample from the large nursing service “Deutsche Fachpflege” included more than 2.000 individuals receiving HIC between January and December 2025. The underlying disease affected the nervous system or muscles in approximately 81% and the lungs or tracheobronchial system in approximately 16% (3). Twenty-five percent needed tracheostomy invasive ventilation, and 7% required noninvasive ventilation, 58% did not need mechanical ventilation, and in 10% no data on mechanical ventilation were reported (3). Forty two percent received HIC at home, 54% in shared appartements, and 3% in specialized nursing homes (3).

Available data are therefore incomplete or prone to selection bias, and do not discriminate between HIC at home and in institutions. The current study’s objective was to characterize individuals receiving home-based intensive care from a private German provider to inform healthcare structures and clinical practice guidelines.

## Methods

On June 12, 2024, the quality management department of a private German home-based intensive care service collected cross-sectional data from all individuals receiving home-based intensive care. The variables were determined by the central quality management department of the home-based intensive care service in collaboration with nursing supervisors (e.g., certified intensive care nurses and respiratory therapists). The selected variables reflect key domains of HIC, including demographic characteristics, place of care, primary diagnoses, tracheal tubes, mechanical ventilation, and other life-supporting therapies, which are central to quality assessment, reimbursement procedures, and care planning in this field. Data on age, gender, and place of care as well as clinical routine data were exported from the routine nursing documentation software MediFox ambulant – Pflegedokumentationssoftware, MEDIFOX DAN GmbH, Hildesheim, Germany. Primary diagnoses were derived from routine nursing documentation in the MediFox ambulant system, which is based on medical discharge summaries and physician reports available to the nursing services. Nursing supervisors reviewed and categorized the documented primary diagnoses into four predefined groups (pulmonological, neurological, malignant, and infectious diseases). These diagnostic categories represent the most common underlying conditions in HIC. They are closely linked to care complexity, and they align with regulatory and quality assurance frameworks in Germany. The recorded data underwent a plausibility check by responsible personnel from the respective care services. The information recorded by the system was compared with the actual care situation of the individuals, checked for logical consistency, and incomplete or contradictory entries were supplemented or corrected. Descriptive statistical analysis was performed using counts and percentages as only categorical variables were recorded.

## Results

Data from 851 individuals were collected. There were no missing data unless reported otherwise. Three hundred and thirty two (39%) were female and 519 (61%) were male. Hundred and fifty one (18%) were younger than 40 years, 65 (8%) were 40 to 49 years, 123 (14%) 50 to 59 years, 244 (29%) 60 to 69 years, 181 (21%) 70 to 79 years, 84 (10%) 80 to 89 (10%), and three were older than 90 years. Six hundred and eighty two individuals (80%) suffered from various neurological diseases, most frequently intracranial hemorrhage, ischemic stroke and hypoxic encephalopathy. Hundred and sixteen individuals (14%) suffered from COPD, and 30 (4%) from oral, pharyngeal or laryngeal cancer. Twenty seven (3%) had been admitted to HIC after a COVID-19 infection (see Table 1). Seven hundred and forty one individuals (89%) were tracheostomized, of whom 220 (26%) needed invasive Fourty four (5%) required noninvasive ventilation, 14 (2%) hemodialysis, and four a left ventricular assist device (LVAD).

**Table 1:** Patients diagnoses (percentages relate to the total of *n* = 851 patients, multiple diagnoses possible).

Category	Variable	Females (*n* = 332)	Males (*n* = 519)	Total (*n* = 851)
Neurological conditions	Intracranial hemorrhage	66 (8%)	75 (9%)	141 (17%)
	Hypoxic encephalopathy	40 (5%)	64 (8%)	104 (12%)
	Ischemic stroke	35 (4%)	60 (7%)	95 (11%)
	Traumatic brain injury	11 (1%)	44 (5%)	55 (6%)
	Motor neuron disease	14 (2%)	36 (4%)	50 (6%)
	Vegetative state	21 (2%)	20 (2%)	41 (5%)
	Spinal cord injury	8 (1%)	12 (1%)	20 (2%)
	Multiple sclerosis	8 (1%)	8 (1%)	16 (2%)
	Other	66 (8%)	94 (11%)	160 (19%)
Respiratory diseases	COPD	45 (5%)	71 (8%)	116 (14%)
	Secondary lung impairment	30 (4%)	53 (6%)	83 (10%)
	Other respiratory conditions	10 (1%)	11 (1%)	21 (2%)
Oncological diseases	Oral, pharyngeal or laryngeal cancer	3 (0%)	27 (3%)	30 (4%)
	Other malignancy	4 (0%)	9 (1%)	13 (2%)
Infectious diseases	Covid-19	10 (%)	17 (%)	27 (3%)
	Other infectious diseases	1 (0%)	5 (1%)	6 (1%)

**Figure 1:**
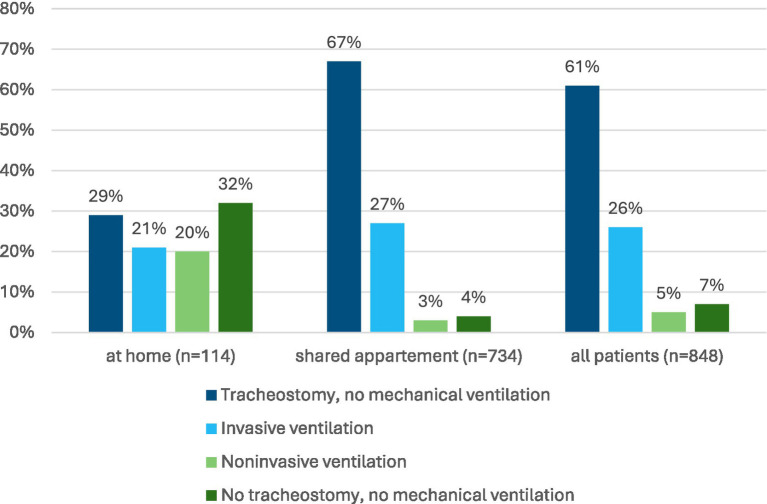
Comparison of patients living at home vs. shared appartements with respect to the prevalence life-supporting technologies (*n* = 3 patients excluded because of missing data).

Seven hundred and thirty four (86%) individuals were living in shared apartments and 117 (14%) lived at home with 24 h individual nursing care. The proportion of tracheostomized individuals in general, and the proportion of individuals with tracheostomy invasive ventilation was higher in shared appartements while the proportion of non-tracheostomized individuals in general, and the proportion of individuals with non-invasive ventilation was higher in HIC at home (see Figure 1).

A comparison of the most frequent primary diagnoses with respect to the prevalence of tracheostomy and mechanical ventilation as well as the place of care are shown in Table 2. Comparisons related to neurological syndromes (e.g. “vegetative state”), secondary pulmonary impairment and non-classified (“other”) diagnoses are not reported. All individuals with oral, pharyngeal or laryngeal cancer were tracheostomized, while tracheostomy was least prevalent in motor neuron disease. Non-invasive ventilation and invasive ventilation via tracheostomy were more prevalent in COPD and motor neuron disease than in other diagnostic groups. The proportion of individuals receiving HIC at home was highest in motor neuron disease.

**Table 2:** Comparison of patients with the most frequent primary diagnoses with respect to the prevalence life-supporting technologies.

Variable	ICH	COPD	HE	IS	TBI	MND	OPLC
Tracheostomy, no mechanical ventilation	118 (84%)	42 (36%)	84 (81%)	77 (81%)	46 (84%)	5 (10%)	27 (90%)
TIV	19 (13%)	65 (56%)	17 (16%)	15 (16%)	5 (9%)	33 (66%)	3 (10%)
NIV	0 (0%)	8 (7%)	1 (1%)	2 (2%)	0 (0%)	8 (16%)	0 (0%)
HIC at home	8 (6%)	2 (2%)	8 (8%)	4 (4%)	4 (7%)	7 (14%)	1 (3%)
HIC in s. appart.	133 (94%)	114 (98%)	96 (92%)	91 (96%)	51 (93%)	43 (86%)	29 (97%)
Total (100%)	141	116	104	95	55	50	30

TIV, tracheostomy invasive ventilation; NIV, non-invasive ventilation; HIC, home-based intensive care; ICH, intracranial hemorhage; s. apart., shared appartement; HE, hypoxic encephalopathy; IS, ischemic stroke; TBI, traumatic brain injury; MND, motor neuron disease; OPLC, oral, pharyngeal or laryngeal cancer.

## Discussion

This study reports cross-sectional data on places of care and demographic and clinical characteristics from a large sample. Demographic data are consistent with what has been reported from three samples cited in the introduction: The individuals´ age covers the whole life span with about every fifth individual being younger than 40 years, the majority being 60 years or older, but only a few being 90 years old or older. Individuals requiring HCI are predominantly male (3, 6, 7). As demonstrated in Table 1 this relates to all diagnoses and syndromes except for multiple sclerosis and vegetative state where the distribution is even. Although some of the neurological diagnoses registered in this study are more prevalent in men than in women the causes of the observed gender distribution are likely more complex (8, 9). Disease severity, patient journey and preferences of the individuals and their relatives or legal representatives also matter. Men and their relatives or legal representatives may be more likely to opt for life-supporting technologies and subsequent HIC (10).

According to our data and the data of another large nursing service individuals are more often cared for in shared appartements than at home, but reports based on health insurance data or the institution controlling the entitlement to HCI indicate that individuals are more often cared for at home (3, 6, 7). This may reflect organizational characteristics of the nursing services from which the data were collected. Additionally, the healthcare landscape of HCI is evolving and the predominant places of care may change (6). The proportion of individuals with tracheostomy in general and with tracheostomy invasive ventilation in particular is higher in shared appartements but the proportion of individuals with non-invasive ventilation is higher at home. Also, a higher proportion of individuals with motor neuron disease receives HIC at home when compared to other diseases. In HIC at home chances for equal participation may be better but institutionalized care may be more adapted to the use of life supporting technologies.

Neurological diseases lead to complex sequelae: In cerebral diseases dysphagia and cough insufficiency are frequently found while ventilatory failure is rarely seen, e.g., in acquired central hypoventilation syndrome resulting from lesions of the lateral medulla oblongata (11). Individuals with cervical spinal cord injury and neuromuscular disease are prone to ventilatory pump failure, cough insufficiency and dysphagia. Not only in neurological diseases but also in COPD ventilatory pump failure, dysphagia and cough insufficiency occur (12). While individuals with acquired central hypoventilation syndrome or ventilatory pump failure require mechanical ventilation, individuals with dysphagia or cough insufficiency may require tracheostomy (13). Consecutively the high prevalence of neurological diseases and COPD results in a high proportion of individuals with tracheostomy in general and with tracheostomy invasive ventilation in particular in HIC. Additionally, all individuals with oral, pharyngeal or laryngeal cancer in this study were tracheostomized. However, the proceedings of the pre-treating intensive care units, weaning units, and early rehabilitation units may influence the prevalence of tracheostomy and invasive ventilation, too. As the prognosis of individuals with COPD and tracheostomy invasive ventilation is comparably poor and non-invasive ventilation is recommended for COPD and for neuromuscular diseases in the German clinical practice guidelines the high prevalence of tracheostomy has to be addressed (14, 15).

This study documents that HIC is specialized in care for individuals with tracheostomy in general and tracheostomy invasive ventilation in particular. Neurological diagnoses are most prominent, but COPD is also relevant in HIC. Individuals requiring HIC need multidisciplinary care by different medical specialists and other healthcare professions, rehabilitation, palliative care, and sometimes weaning from mechanical ventilation and decannulation (4, 16, 17). Telemedicine is currently evolving as a promising approach to foster weaning from mechanical ventilation and decannulation (18). There are specialized multidisciplinary care inpatient healthcare structures that are suitable for treatment of individuals requiring HIC: (a) institutions specialized in weaning from mechanical ventilation certified by the German Society for Pulmonology and Mechanical Ventilation (DGP) or the German Society for Anaesthesiology and Intensive Care (DGAI), (b) centers for weaning in neurological-neurosurgical early rehabilitation certified by the German Society for Neurorehabilitation (DGNR), (c) centers for individuals with spinal cord injury certified by the German-speaking Medical Society for Paraplegiology (DMGP), and (d) other institutions providing early rehabilitation (19). The benefits of intersectoral healthcare for individuals requiring HIC are currently under investigation (20). In addition, some medical centers for adults with disabilities are able to provide multidisciplinary outpatient care to individuals requiring HIC. The relatively new model of mobile rehabilitation with rehabilitation provided at the place of care may also be suitable for individuals requiring HIC (21). A continuum-of-care with barrier-free access to the aforementioned inpatient and outpatient healthcare structures may offer the best rehabilitative outcomes for individuals requiring HIC. This will be addressed in a clinical practice guideline regarding medical, therapeutic and nursing care for individuals with diseases of the nervous system or the muscles requiring HIC (22). Future research is needed to understand regional differences in healthcare structures for individuals requiring HIC, to analyse the patient journey, to identify factors influencing survival and other relevant outcomes, e.g., by building up a prospective patient registry.

### Limitations

There are several limitations to this study. First of all, only one predefined diagnosis could be chosen per category (pulmonological, neurological, malignant and infectious disease). This may have led to underreporting, when there were more diseases present from one category. Also, certain comorbidities were not recorded at all, e.g., cardiac, renal or hepatic disease. This again may have resulted in an underestimation of disease complexity. Additionally, some diagnoses were syndromes (e.g., vegetative state) rather than diseases, and data related to diagnoses could not be compared with data related to syndromes. Additionally, the age groups presented in this study do not allow to differentiate between children, adolescents and young adults, which again limits conclusions regarding specific treatment needs of this population.

## Conclusion

In Germany, neurological diagnoses and COPD are predominant in HIC. Most individuals are tracheostomized, and one in four requires invasive ventilation via tracheostomy. They require multidisciplinary care by neurologists, pulmonologists, specialist nurses, respiratory therapists, speech-and-language therapists, physiotherapists, occupational therapists, and other professionals. Neurological rehabilitation of individuals needing HIC should be established, accompanied by research.

## Data Availability

The raw data supporting the conclusions of this article will be made available by the authors, without undue reservation.
